# Prevalence and genetic diversity of endosymbiotic bacteria infecting cassava whiteflies in Africa

**DOI:** 10.1186/s12866-015-0425-5

**Published:** 2015-05-02

**Authors:** Saptarshi Ghosh, Sophie Bouvaine, MN Maruthi

**Affiliations:** Natural Resources Institute, University of Greenwich, Chatham, ME4 4 TB Kent UK

**Keywords:** Cassava, Whitefly, mtCOI, *Wolbachia*, *Rickettsia*, *Arsenophonus*

## Abstract

**Background:**

Cassava provides over half of the dietary requirement for more than 200 million poor in Africa. In recent years, cassava has been affected by an epidemic of a virus disease called cassava brown streak disease (CBSD) that is spreading in much of eastern and central Africa, affecting food security and the economic development of the poor. The viruses that cause CBSD are transmitted by the insect vector whitefly (*Bemisia tabaci*), which have increased to very high numbers in some African countries. Strains of endosymbiotic bacteria infecting whiteflies have been reported to interact specifically with different whitefly populations with varied effects on its host biology and efficiency of virus transmission. The main aim of this study was therefore to investigate the prevalence and diversity of the secondary endosymbiotic bacteria infecting cassava whiteflies with a view to better understand their role on insect population dynamics and virus disease epidemics.

**Results:**

The genetic diversity of field-collected whitefly from Tanzania, Malawi, Uganda and Nigeria was determined by mitochondrial DNA based phylogeny and restriction fragment length polymorphism. Cassava in these countries was infected with five whitefly populations, and each one was infected with different endosymbiotic bacteria. Incidences of *Arsenophonus*, *Rickettsia*, *Wolbachia* and *Cardinium* varied amongst the populations. *Wolbachia* was the most predominant symbiont with infection levels varying from 21 to 97%. Infection levels of *Arsenophonus* varied from 17 to 64% and that of *Rickettsia* was 0 to 53%. *Hamiltonella* and *Fritschea* were absent in all the samples. Multiple locus sequence typing identified four different strains of *Wolbachia* infecting cassava whiteflies. A common strain of *Wolbachia* infected the whitefly population Sub-Saharan Africa 1-subgroup 1 (SSA1-SG1) and SSA1-SG2, while others were infected with different strains. Phylogeny based on 16S rDNA of *Rickettsia* and 23S rDNA of *Arsenophonus* also identified distinct strains.

**Conclusions:**

Genetically diverse bacteria infect cassava whiteflies in Africa with varied prevalence across different host populations, which may affect their whitefly biology. Further studies are required to investigate the role of endosymbionts to better understand the whitefly population dynamics.

**Electronic supplementary material:**

The online version of this article (doi:10.1186/s12866-015-0425-5) contains supplementary material, which is available to authorized users.

## Background

The whitefly, *Bemisia tabaci* (*Hemiptera*: *Aleyrodidae*) has gained importance as one of the most important agricultural pests owing to its wide geographic spread, large host range of over 500 hosts, and most significantly as a vector of over 100 different plant viruses in the tropical and subtropical regions of the world [1,2]. *B. tabaci* is a cryptic species complex comprising at least 24 morphologically indistinguishable species [3] with a proposed origin in sub-Saharan Africa (SSA) and with high variability in mitochondrial cytochrome oxidase I (mtCOI) nucleotide sequences amongst major geographical clades [2,4]. Cassava, a key food security crop throughout SSA, suffers devastating yield losses due to *B. tabaci*-borne cassava mosaic begomoviruses (CMBs) and cassava brown streak viruses (CBSVs). These cause cassava mosaic disease (CMD) and cassava brown streak disease (CBSD), respectively [5,6]. Five genetically distinct groups of *B. tabaci,* named Sub-Saharan Africa 1 to 5 (SSA 1–5) colonise cassava in SSA. These have been generally referred to as cassava whiteflies in this and other studies. SSA1 occurs throughout the SSA, SSA2 in East and West Africa, SSA3 in Cameroon and Togo, SSA4 only in Cameroon and SSA5 in South Africa [6]. Based on mtCOI sequence divergences, SSA1 was further divided into four subgroups; SSA1- subgroup 1 (SSA1-SG1), SSA1-SG2, SSA1-SG3 and SSA1-SG4 [6].

Superabundant *B. tabaci* populations, commonly numbering more than 1000 adults per top five leaves, of cassava plants, have been associated with the rapid spread of CMD pandemic in East and Central Africa since the late 1990s. SSA2, the then super abundant population which was also described as the ‘invader or UG2’, was associated with the spread of the CMD pandemic [7,8]. In recent years, a shift in the *B. tabaci* population has occurred with the relative frequency of SSA1-SG1 increasing from 24.6% to 89.2%, while the frequencies of SSA2 and SSA1-SG2 decreasing significantly from 63.9% to 1.4%, and 11.5% to 1.4%, respectively, between 1997 and 2010 [6]. The reasons for this natural shift in cassava population remain unknown. Similar shift in genetic diversity of populations are reported for *B. tabaci* species, mainly with respect to the population replacement by the invasive Middle East-Asia Minor 1 (MEAM1, previously B-biotype) and Mediterranean (MED, previously Q-biotype) populations [9-11].

In addition to the primary endosymbiont *Portiera aleyrodidarum*, the species *B. tabaci* has been reported to harbour six vertically transmitted secondary endosymbionts, *Arsenophonus*, *Wolbachia*, *Hamiltonella*, *Cardinium*, *Fritschea* and *Rickettsia* [12-14]. Recently, a new bacterium named *Candidatus hemipteriphilus asiaticus* was also found to infect *B. tabaci* from China [15]. Several of these endosymbionts can affect the biology and behaviour of *B. tabaci. Wolbachia* and *Cardinium* in particular are known to induce cytoplasmic incompatibility (CI), a process in which the host reproduction is manipulated to allow rapid spread of bacteria through insect populations [16]. Whether such phenotypes are induced by these bacteria in *B. tabaci* remains unknown. *Rickettsia*, when infecting MEAM1, provided fitness benefits by increased fecundity and survival [17], increased heat stress tolerance [18], defence against pathogens [19] but occasionally also increased the susceptibility to insecticides [20]. *Fritschea* has reported negative impact with reduced fecundity and narrowing the host range of infected New World species of whiteflies [21]. Endosymbionts can also alter the vector ability of *B. tabaci. Hamiltonella* in MEAM1 and *Arsenophonus* in Asia II populations facilitated virus transmission by releasing a bacterial chaperonin GroEL that binds and protects virus particles during their transit through the insect body [22,23]. *Hamiltonella* in the MED and *Rickettsia* in MEAM1 populations are also reported to increase acquisition, retention and transmission of *Tomato yellow leaf curl virus* [24,25]. The study of intracellular bacterial communities in these whiteflies and their impact on the host was essential for understanding the dynamics of insect populations and their vector abilities. In this study, we identified the endosymbionts infecting cassava whiteflies, determined their infection frequencies in different populations and characterised the diverse bacterial species by sequencing. We have also developed a cost effective and reliable restriction fragment length polymorphism (RFLP) diagnostic method for the molecular typing of the cassava whitefly populations.

## Results

### RFLP for molecular typing of cassava whiteflies

**Figure 1:**
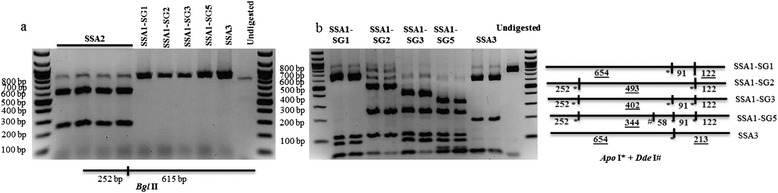
Detection of cassava whitefly populations based on RFLP profiles for high throughput screening. **a**: Detecting SSA2 by digestion with *Bgl* II, **b**: Detecting SSA1 and SSA3 by *Apo* I and *Dde* I. Underlined values represent the diagnostic fragments for the respective whitefly populations.

The mtCOI locus has been the most commonly used marker for genotyping whiteflies but the cost and time involved in gene sequencing and analysis are a limiting factor for routine diagnosis and processing large number of samples in epidemiological studies. We therefore developed a quick and cost-effective RFLP technique as an alternative to type SSA cassava whiteflies used in this study that efficiently identified the different populations. The RFLP was carried out in two steps. In the first step, digesting mtCOI products with *Bgl* II cleaved SSA2 into two fragments of size 615 and 252 bp but did not cleave mtCOI loci from other populations (Figure 1a). In the second step, digesting mtCOI products from SSA1 and SSA3 with *Apo* I and *Dde* I produced 2 to 5 fragments of distinctive sizes (Figure 1b). SSA1-SG1 and SSA3 were distinguished by the presence of fragments 122 and 213 bp, respectively. SSA1-SG2, SSA1-SG3 and SSA1-SG5 were identified by the presence of bigger fragments of 493, 402 and 344 bp, respectively (Figure 1b). These patterns were obtained consistently on 20 samples digested for each population. Fragments below 100 bp size were not visualised reliably on agarose gels, which were therefore discounted from the analysis.

### Cassava whitefly diversity and detection

**Figure 2:**
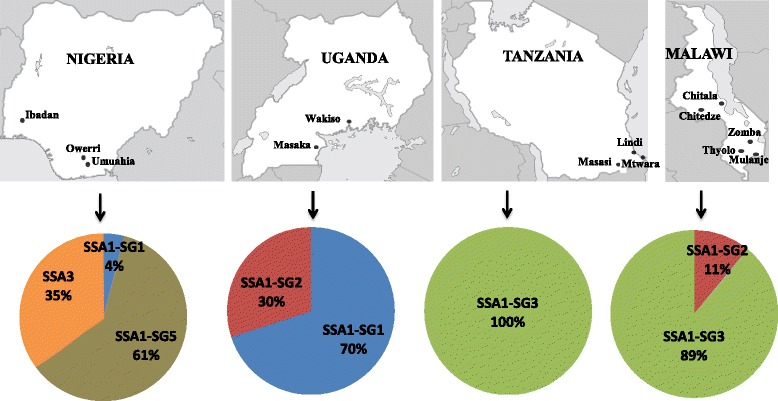
Frequency of *B. tabaci* populations found in the four sampled countries.

**Figure 3:**
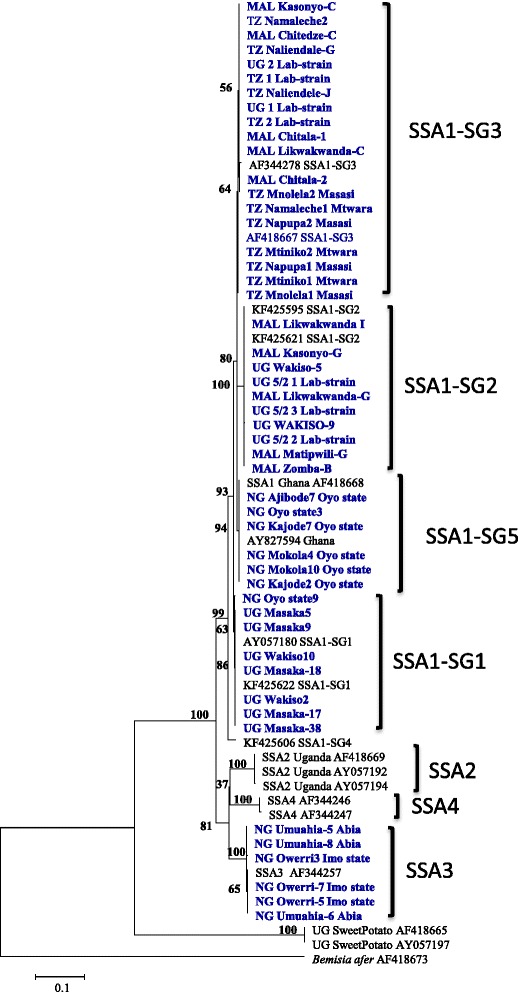
Phylogeny of mtCOI nucleotide sequences (697 bp) of *B. tabaci* infesting cassava together with reference sequences from Genbank. Genbank accession numbers for the submitted sequences are KM377899 to KM377952, and KM407138 to KM407141.

The mtCOI locus of cassava whiteflies indicated the predominance of SSA1 populations in the countries sampled, the only other group present was SSA3 in Nigeria. All *B. tabaci* samples analysed from Tanzania (35 out of 35) belonged to SSA1-SG3 type. In Malawi, about 89.1% (41/46) whiteflies were SSA1-SG3 and the remaining 10.8% (5/46) were SSA1-SG2. In Uganda, 69.4% (68/98) were SSA1-SG1 and 30.6% (30/98) were SSA1-SG2 (Figure 2). The Nigerian (Ibadan) populations belonged to the SSA1 group in the phylogenetic trees but did not cluster with any of the known four sub-groups. They clustered separately with sequences from Ghana from the database; they are therefore referred to as SSA1-SG5 (Figure 3). In Nigeria, 60.3% (41/68) were SSA1-SG5, 35.3% (24/68) SSA3 and 4.4% (3/68) were SSA1-SG1 type (Figure 2). SSA2 and SSA1-SG4 were not found in our study.

### Prevalence of bacterial endosymbionts

**Figure 4:**
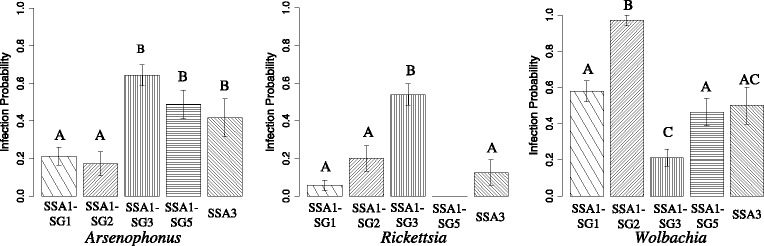
Mean infection probabilities of symbionts in the five cassava whitefly populations as determined by simple binomial logistic regression. Mean infection probability of a symbiont within the populations was compared by Tukey’s HSD test and significant difference is indicated by different letters.

The primary endosymbiont *Portiera* was detected in all the samples as expected. The secondary symbionts were found in 77.3% (191 whiteflies infected out of 247 tested) of the insects and their prevalence varied significantly across the different whitefly populations (Figure 4). The overall infection frequencies of *Wolbachia*, *Arsenophonus*, *Rickettsia* and *Cardinium* in the cassava whiteflies were 49.4% (122/247), 40.5% (100/247), 22.3% (55/247) and 0.8% (2/247), respectively. *Hamiltonella* and *Fritschea* were not detected in any of the whiteflies tested.

**Figure 5:**
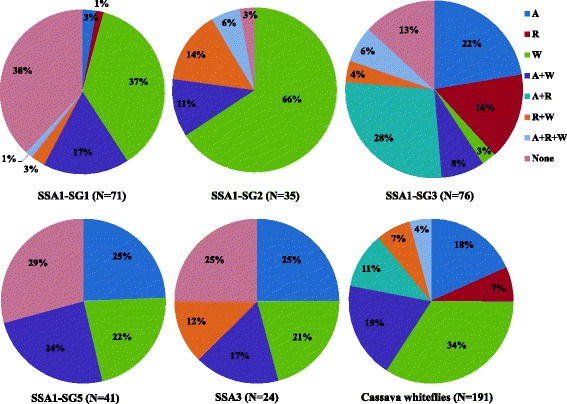
Pattern of infections of symbionts in different whitefly populations. Alphabets represent infection by each symbiont, A = *Arsenophonus*, R = *Rickettsia*, W = *Wolbachia*, C = *Cardinium*, None = free of secondary endosymbionts).

Highest and lowest rates of infection by *Arsenophonus* were seen in SSA1-SG3 (64.5%, 49/76) and SSA1-SG2 (17.1%, 6/35), respectively (see Additional file 1: Table S2). *Arsenophonus* was present mostly as double infections, with *Wolbachia* in SSA1-SG1 (17%) and SSA1-SG2 (11%), and with *Rickettsia* in SSA1-SG3 (28%). *Arsenophonus* was present in SSA1-SG5 and SSA3 mainly as single infections (Figure 5).

*Rickettsia* was absent in SSA1-SG5 but most abundant in SSA1-SG3 (53.9%, 41/76) followed by SSA1-SG2 (20%, 7/35). Its infection levels in other populations were negligible. *Cardinium* was the least prevalent endosymbiont, detected only in 2 out of the 76 SSA1-SG3 (2.6%) but not in other populations.

*Wolbachia* was most abundant amongst the secondary bacteria and was the commonest symbiont in SSA1-SG1 and SSA1-SG2 populations, mostly as single infections (Figure 5). It was nearly fixed in SSA1-SG2 (97.1%, 34/35), and was much higher compared to infections seen in all other populations (see Additional file 1: Table S2).

A high percentage of whiteflies were completely free of secondary symbionts in SSA1-SG1 (38.0%) followed by SSA1-SG5 (29.2%), SSA3 (25.0%), SSA1-SG3 (13.1%), and only 2.8% in SSA1-SG2 (Figure 5). Cassava whiteflies predominantly were singly infected by a symbiont (59.1%, 113/191), mostly by *Wolbachia* (34.0%, 65/191) whereas only 36.6% (70/191) and 4.1% (8/191) had double and triple infections, respectively. Co-infections were commonest in SSA1-SG3 (54.5%, 36/66) (Figure 5).

### Genetic diversity of endosymbionts

All five MLST fragments were amplified from *Wolbachia* infections from SSA1-SG2 and SSA1-SG3 from East African *B. tabaci* populations. However, only *coxA* was amplified from SSA1-SG5 and none from SSA3 from Nigeria despite exhaustive efforts. For SSA1-SG1, only *coxA*, *ftsZ*, *gatB* and *hcpA* were amplified except for one sample for which all five MLST markers were amplified.

**Figure 6:**
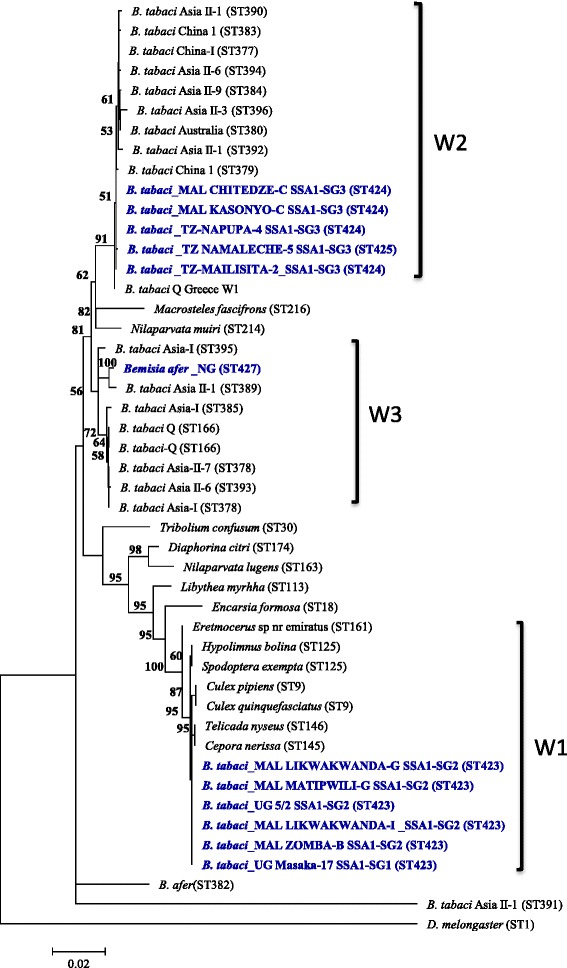
Phylogeny of concatenated MLST (2079 bp) nucleotide sequences of *Wolbachia* infecting whiteflies and other insect species*.* Strain names in the parentheses indicate the various *Wolbachia* sequence types.

**Figure 7:**
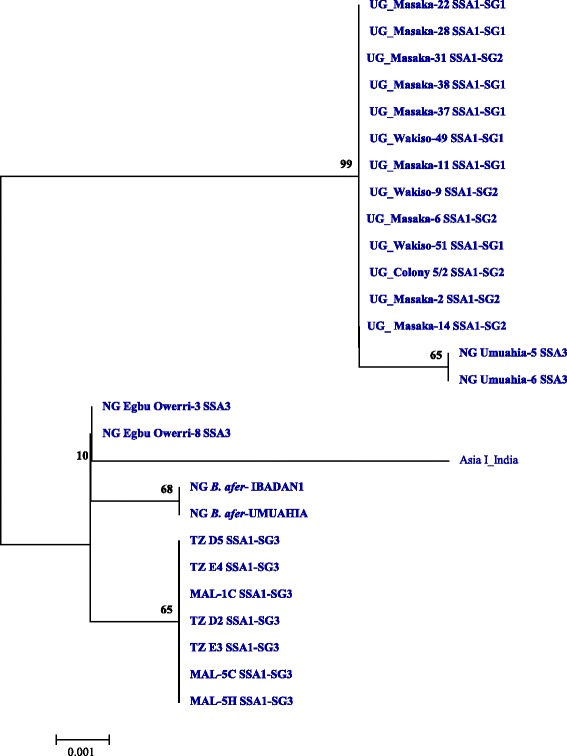
Phylogeny of *Wolbachia wsp* (596 bp) nucleotide sequences infecting cassava whiteflies in sub-Saharan Africa. Genbank accession numbers for submitted sequences are KP208705 to KP208733.

Four unique *Wolbachia* sequence types were identified in this study, which were all submitted to the *Wolbachia* pubMLST database (Table 1). SSA1-SG1 and SSA1-SG2 were infected with identical *Wolbachia* based on five MLST alleles. These were unique to African cassava whiteflies as they shared no allele from other reported *Wolbachia* from *B. tabaci* but shared four common alleles with *Eretmocerus* sp. (parasitoid of whitefly), butterflies and *Spodoptera exempta* from wide geographical distances in the USA, Japan, India and Tanzania (Table 1). In contrast, SSA1-SG3 was infected with two different *Wolbachia*, but they shared three alleles (*coxA* = 88, *hcpA* = 106, *fbpA* = 9) with *B. tabaci* from China and USA. Phylogeny of the concatenated MLST sequences of *Wolbachia* from whiteflies clustered into three sub-clades, W1, W2 and W3 (Figure 6). W1 sequences were from SSA1-SG1 and SSA1-SG2, and these were closely related (≥99.9% identical, Table 2) to *Culex* and butterfly species (*Hypolimnus*, *Cepora* and *Telicada*). W2 isolates contained SSA1-SG3, and was closer to *Wolbachia* from *B. tabaci* from other geographical regions and host plants. W3 consisted of isolates from *B. tabaci* from Asia and *Bemisia afer* from Nigeria. Similar results were obtained when the phylogenetic analysis of the *wsp* gene was sequenced for *Wolbachia* as the SSA1-SG1 and SSA1-SG2 were clustered together and separately from SSA1-SG3 (Figure 7). Comparison of *Wolbachia* strains showed that W1 isolates differed by a minimum of 4.5% nucleotides from W2 and W3 isolates, and W2 and W3 isolates differed by a minimum of 1% for MLST sequences (Table 2).

**Table 1:** **Comparison of MLST profile of** ***Wolbachia*** **from cassava** ***B. tabaci*** **with those from the pubMLST database, specimens in bold were generated in this study**

**Host**	**Super group**	**Country**	***coxA***	***fbpA***	***ftsZ***	***gatB***	***hcpA***	**Sequence type**
***B. tabaci*** **(SSA1-SG1)**	B	Uganda	14	4	73	4	3	423*
***B. tabaci*** **(SSA1-SG2)**	B	Malawi, Uganda	14	4	73	4	3	423*
***B. tabaci*** **(SSA1-SG3)**	B	Tanzania, Malawi	88	9	105	9	106	424*
***B. tabaci*** **(SSA1-SG3)**	B	Tanzania	88	404*	105	9	106	425*
***B. tabaci*** **(SSA1-SG5)**	B	Nigeria	88	----	-----	-----	----	
***B. afer***	B	Nigeria	88	89	198*	105	106	427*
*B. tabaci* (MED)	B	USA	88	165	7	105	106	166
*B. tabaci* (China I)	B	China	88	9	170	207	13	377
*B. tabaci* (Asia II 1)	B	China	88	390	170	207	234	391
*B. tabaci* (China 1)	B	China	88	9	170	105	13	379
*B. tabaci* (Asia II 7)	B	China	88	387	7	105	106	378
*B. tabaci* (Asia 1)	B	China	88	387	182	207	106	395
*B. tabaci* (Australia)	B	Australia	88	9	170	207	221	380
*B. tabaci* (Asia II 9)	B	China	88	386	170	207	13	384
*Eretmocerus sp. nr. emiratus*	B	USA	14	4	73	105	3	161
*Hypolimnus bolina*	B	Japan	14	4	73	4	40	125
*Telicada nyseus*	B	India	14	4	73	4	40	125
*Spodoptera exempta*	B	Tanzania	14	4	73	4	40	125
*Cepora nerissa*	B	India	14	4	36	4	3	145

‘*’New additions of *Wolbachia* sequence types to the database by this study, and ‘----’ failure to amplify genes in PCR amplifications.

**Table 2:** **Percentage nucleotide identities of selected** ***Wolbachia*** **strains based on concatenated MLST sequences (p-distances were calculated using MEGA 5.0)**

**UG-5/2_SSA1-SG2 (423)**																				
**MAL_SSA1-SG2 (423)**	100																			
**UG_SSA1-SG1 (423)**	100	100																		
*H bolina* (125)	99.95	99.95	99.95																	
*C pipiens*_(9)	99.90	99.90	99.90	99.86																
*Eretmocerus *(161)	99.71	99.71	99.71	99.66	99.61															
**MAL_SSA1-SG3 (424)**	95.56	95.56	95.56	95.61	95.47	95.85														
**TZ_SSA1-SG3 (424)**	95.56	95.56	95.56	95.61	95.47	95.85	100													
**TZ_SSA1-SG3 (425)**	95.51	95.51	95.51	95.56	95.42	95.80	99.95	99.95												
China-I (377)	95.47	95.47	95.47	95.51	95.37	95.75	99.90	99.90	99.86											
Asia_II-3 (396)	95.27	95.27	95.27	95.32	95.18	95.56	99.61	99.61	99.57	99.71										
Asia_II-9 (384)	95.42	95.42	95.42	95.47	95.32	95.71	99.86	99.86	99.81	99.95	99.76									
Australia (380)	95.47	95.47	95.47	95.51	95.37	95.75	99.86	99.86	99.81	99.95	99.76	99.90								
***B afer*** **_Nigeria (427)**	96.53	96.53	96.53	96.58	96.43	96.82	98.22	98.22	98.17	98.12	97.88	98.07	98.07							
MED (166)	96.19	96.19	96.19	96.24	96.09	96.48	98.99	98.99	98.94	98.89	98.65	98.84	98.84	99.23						
Asia_II-1 (389)	96.24	96.24	96.24	96.29	96.14	96.53	98.22	98.22	98.17	98.31	98.02	98.26	98.26	99.61	99.04					
Asia_II-6 (393)	96.09	96.09	96.09	96.14	96.00	96.38	98.99	98.99	98.94	98.89	98.65	98.84	98.84	99.13	99.90	98.94				
Asia-II-7 (378)	96.14	96.14	96.14	96.19	96.04	96.43	99.04	99.04	98.99	98.94	98.70	98.89	98.89	99.18	99.95	98.99	99.95			
*B afer* (382)	95.18	95.18	95.18	95.22	95.18	95.47	96.62	96.62	96.58	96.53	96.29	96.48	96.48	96.33	95.90	96.04	95.80	95.85		
*D melongaster* (1)	88.08	88.08	88.08	88.13	87.99	88.23	89.10	89.10	89.05	89.10	88.95	89.05	89.05	88.47	88.86	88.47	88.86	88.90	88.95

**Figure 8:**
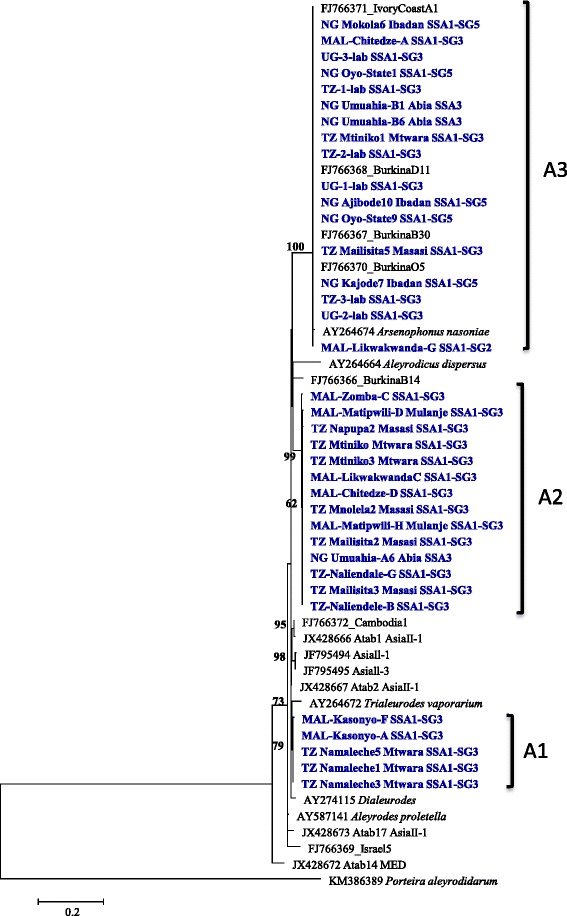
Phylogeny of *Arsenophonus* infecting whitefly species based on 23S rDNA (401 bp) nucleotide sequences. Genbank accession numbers for the submitted sequences are KM377863 to KM377898.

The 23S rDNA sequences of *Arsenophonus* from cassava whiteflies clustered into three sub clades A1, A2, A3 with bootstrap scores of >70% (Figure 8). A3 isolates differed by 5.8% from A1, and 9.4% from A2 isolates (Table 3). These were incongruent with the evolution of the whitefly host based on mtCOI phylogeny. The samples belonging to clade A3 had additional 160 bp sequences and closely related (99.5% identity, Table 3) to sequences from *Arsenophonus nasoniae,* a male killing endosymbiont in the parasitic wasp, *Nasonia vitripennis*. One SSA1-SG2 and SSA1-SG3 sample was each infected by both A2 and A3 strains of *Arsenophonus*.

**Table 3:** **Percentage nucleotide identities of 23S rDNA sequences of** ***Arsenophonus*** **strains (p-distances were calculated using MEGA 5.0)**

**TZ_Namaleche1_SSA1-SG3**															
**TZ_Namaleche3_SSA1-SG3**	100														
**MAL-Kasonyo-F_SSA1-SG3**	99.8	99.8													
**TZ-Naliendale-B_SSA1-SG3**	95.4	95.4	95.2												
**NG_Umuahia-A6_SSA3**	95.4	95.4	95.2	100											
**MAL-Zomba-C_SSA1-SG3**	95.2	95.2	94.9	99.8	99.8										
**TZ-1-lab_SSA1-SG3**	94.2	94.2	94.0	90.6	90.6	90.8									
**MAL-Chitedze-A_SSA1-SG3**	94.2	94.2	94.0	90.6	90.6	90.8	100								
**NG_Oyo-State1_SSA1-SG5**	94.2	94.2	94.0	90.6	90.6	90.8	100	100							
AY264674_*A_nasoniae*	94.2	94.2	94.0	90.6	90.6	90.8	99.5	99.5	99.5						
FJ766366_ASL_Burkina_Faso	95.7	95.7	95.4	96.4	96.4	96.1	91.1	91.1	91.1	91.1					
FJ766370_MED_Burkina_Faso	94.2	94.2	94.0	90.6	90.6	90.8	100	100	100	99.5	91.1				
JX428666_AsiaII_1	98.1	98.1	97.8	95.9	95.9	95.7	92.8	92.8	92.8	92.8	95.7	92.8			
JF795495_Asiall_3	98.3	98.3	98.1	95.2	95.2	94.9	93.2	93.2	93.2	93.2	95.4	93.2	97.8		
FJ766369_MED_Israel	94.7	94.7	94.4	92.3	92.3	92.0	89.6	89.6	89.6	89.6	92.3	89.6	93.7	94.0	
JX428672_MED_China	91.5	91.5	91.3	91.3	91.3	91.1	87.0	87.0	87.0	87.0	90.6	87.0	91.5	91.8	89.4

**Figure 9:**
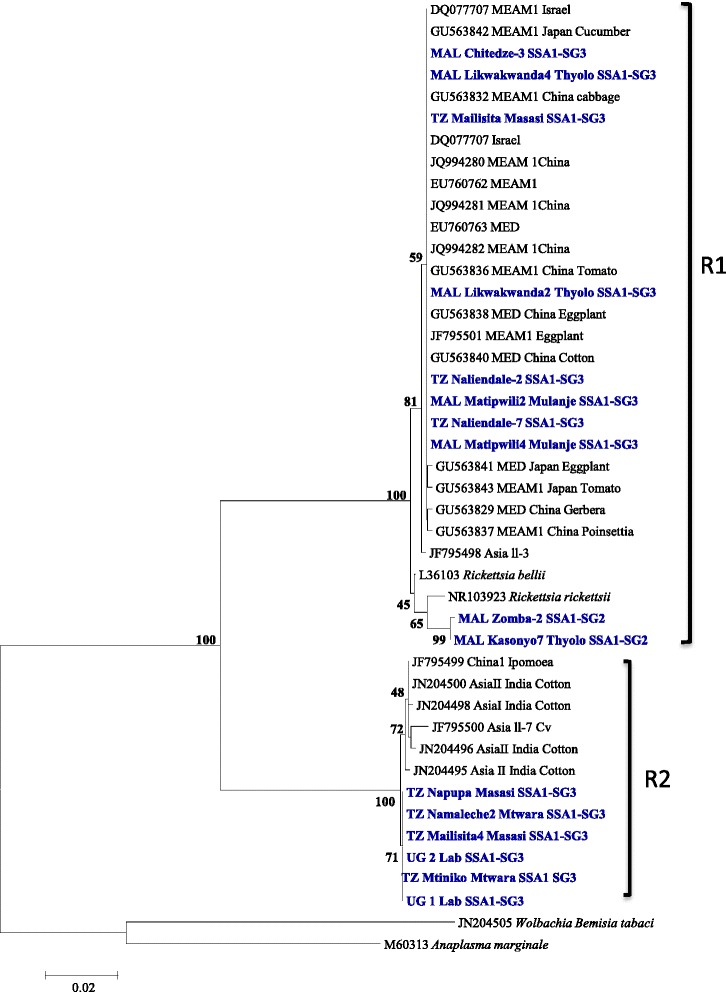
Phylogeny of whitefly-infecting *Rickettsia* 16S rDNA (859 bp) nucleotide sequences. Genbank accession numbers for the submitted sequences are KM386372 to KM38687.

**Figure 10:**
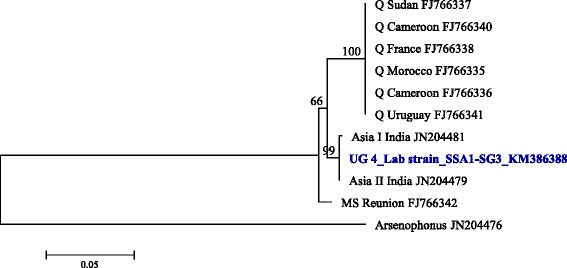
Phylogeny of *Cardinium*, based on the 16S rDNA sequences, infecting whiteflies around the world.

The *Rickettsia* 16S rDNA sequences grouped into two clusters, R1 and R2 (Figure 9) with more than 8.5% nucleotide distances between them (Table 4). R1 strains were detected only in SSA1-SG3 and SSA1-SG2 populations and were identical to the *Rickettsia* from invasive MEAM1 and MED species which were closer to strains from *Rickettsia* sp. nr *Bellii*. R2 strains were identical to the other strains from native whiteflies from India and China. *Cardinium* was detected only in SSA1-SG3 and the sequences were identical to the strains infecting Indian whiteflies (Figure 10).

**Table 4:** **Percentage nucleotide identities of 16S rDNA sequences of** ***Rickettsia*** **strains (p-distances were calculated using MEGA 5.0)**

**TZ_Mailisita-2_SSA1-SG3**																			
**TZ_Naliendale-7_SSA1-SG3**	100																		
**MAL_Chitedze-3_SSA1-SG3**	100	100																	
**MAL_Zomba-2_SSA1-SG2**	98.4	98.4	98.4																
**MAL_Kasonyo-7_SSA1-SG2**	98.5	98.5	98.5	99.9															
DQ077707_MEAM1_Israel	100	100	100	98.4	98.5														
JQ994281_MEAM1_China	100	100	100	98.4	98.5	100													
EU760763_MED	100	100	100	98.4	98.5	100	100												
L36103_*Rickettsia_bellii*	99.5	99.5	99.5	98.8	99.0	99.5	99.5	99.5											
NR103923_*R_rickettsii*	98.5	98.5	98.5	99.0	99.1	98.5	98.5	98.5	99										
JN204498_Asia_I_India	91.5	91.5	91.5	90.6	90.8	91.5	91.5	91.5	91.5	91.2									
JN204495_Asia_II_India	91.6	91.6	91.6	90.8	90.9	91.6	91.6	91.6	91.6	91.3	99.8								
JF795498_Asia_ll_3	99.9	99.9	99.9	98.3	98.4	99.9	99.9	99.9	99.4	98.4	91.6	91.7							
JF795500_Asia_ll_7	91.1	91.1	91.1	90.3	90.4	91.1	91.1	91.1	91.1	90.9	99.4	99.4	91.2						
JF795499_China1	91.5	91.5	91.5	90.6	90.8	91.5	91.5	91.5	91.5	91.2	99.8	99.8	91.6	99.4					
**UG2_Lab_strain_SSA1-SG3**	91.5	91.5	91.5	90.9	91.0	91.5	91.5	91.5	91.5	91.5	99.5	99.5	91.6	99.2	99.8				
**TZ_Mtiniko_SSA1-SG3**	91.5	91.5	91.5	90.9	91.0	91.5	91.5	91.5	91.5	91.5	99.5	99.5	91.6	99.2	99.8	100			
**TZ_Namaleche-2_SSA1-SG3**	91.3	91.3	91.3	90.8	90.9	91.3	91.3	91.3	91.3	91.3	99.4	99.4	91.5	99.1	99.7	99.9	99.9		
**TZ_Napupa_SSA1-SG3**	90.9	90.9	90.9	90.3	90.4	90.9	90.9	90.9	90.9	90.9	98.7	98.7	91.0	98.4	99.0	99.2	99.2	99.1

## Discussion

The main aim of this study was to determine the prevalence and genetic diversity of secondary endosymbionts infecting cassava whiteflies in SSA. Whiteflies harbour multiple bacterial symbionts that play essential roles on insect biology, evolution and virus transmission. Understanding cassava whitefly diversity and the bacterial communities co-existing, within the cassava ecosystem is essential to understand the near extinction of some cassava populations in recent years, or the development of superabundant populations and the resultant epidemics of CMD and CBSD in Eastern and Central African countries in recent years [8,26,27].

At first, the genetic diversity of cassava whiteflies from Uganda, Tanzania, Malawi and Nigeria was studied by mtCOI sequence. This was done to establish the correlation between the prevalence of symbionts in different whitefly populations. Cassava in these countries was colonised by five genetically different whitefly populations. SSA1 and its various sub-groups was predominant in the countries sampled, the only other group present was SSA3 in Nigeria, while SSA2 was not detected. Only SSA1-SG3 was found in coastal Tanzania, while Malawi had high proportions of SSA1-SG3 (89.1%) than Uganda SSA1-SG1 (69.4%) (Figure 2). Based on mtCOI phylogeny, a new population was found in Nigeria, which we referred to as SSA1-SG5 (Figure 3). SSA1-SG5 was predominant (60.3%) in Nigeria, followed by SSA3 (35.3%) and a very few individuals of SSA1-SG1 (4.4%). Overall, these results are concurrent with the previous studies that have also shown high levels of genetic diversity amongst the cassava whitefly populations in SSA [6,27-29].

As seen above and in previous studies, mtCOI is shown to be a reliable marker for separating whitefly species and sub-populations. However, using this as a marker requires sequencing and thus incurs high costs and time. In addition, the threat of the two cassava virus disease pandemics spread by the superabundant *B. tabaci* populations requires simpler monitoring system for effective disease management. We therefore developed a robust RFLP method for typing cassava whiteflies relatively quickly. Using the two-step method and three restriction enzymes described in this study, we were able to reliably assign whiteflies to phylogenetic groups and subgroups found in this study, and thus saving costs as well as time.

Typing the various bacteria infecting these whiteflies, however, proved far more challenging as some of the methods and primers described in the literature did not work initially on cassava whitefly endosymbionts. This was probably because of the high genetic diversity seen in both cassava whiteflies and the various bacteria that infected them. New primers were therefore developed where necessary and the DNA extraction methods and PCR conditions were optimised. Diagnosis of various bacteria confidently was a pre-requisite to understand the genetic diversity of bacteria infecting cassava whiteflies.

Using the above methods, genetically diverse bacteria were found to infect cassava whiteflies in SSA. *Rickettsia*, *Arsenophonus*, *Wolbachia* and *Cardinium* were detected in cassava whiteflies, but not *Hamiltonella* and *Fritschea. Hamiltonella* was also absent in other native whitefly populations in India and China [30,31], but was reported to be present in SSA1 cassava whiteflies from Tanzania [32]. This is contrasting to our study, and we cannot clearly explain the differences between the two studies at this time. Some of the possible explanations, however, include high site to site variation seen in endosymbiont profiles of cassava whiteflies within a country (Tajebe L., pers comm), and that our samples may have been collected coincidentally from *Hamiltonella*-free sites. Other reasons include the low titre of the bacteria in our samples which was beyond the limits of PCR detection, or primer mismatch in PCR reactions. We did obtain unspecific amplification of *Arsenophonus* from *Hamiltonella*-specific primers in initial studies, which indicated primer mismatch. The *Hamiltonella*-specific primers, therefore, should be used with care in future studies, while the *Hamiltonella* quandary between Tajebe et al. [32] and this study remains to be resolved. We used MLST to characterise *Wolbachia*. All five MLST alleles were amplified from all our populations except only *coxA* was amplified from SSA1-SG5 and none from SSA3 after exhaustive efforts. Difficulties in amplification of MLST alleles have been reported previously, and could be due to high variability of these genes or low titres of the symbiont [33,34]. The surface protein *wsp* was therefore used as an alternative marker and this marker also confirmed the high diversity of *Wolbachia* infecting cassava whiteflies.

Overall, about 77.3% of cassava whiteflies were infected with at least one secondary symbiont, while the remaining 22.7% were completely free of the tested bacteria. These results were similar to the incidences of secondary symbionts seen in other *B. tabaci*, which ranged from 78% to 100% [14,30,35,36]. A high percentage of the superabundant SSA1-SG1 from the CMD pandemic areas [32] and SSA1-SG5 whiteflies [37] were also reported to be free of secondary symbionts. Further studies comparing the fecundity and life cycle of bacteria-infected and uninfected cassava whiteflies is essential to understand the reasons behind the development of superabundant whiteflies, and the supposed interactions between symbionts and cassava whiteflies.

Single infections of bacteria were more prevalent (59% of total infections) in cassava whiteflies than double (37%) and triple (4%) infections. This was slightly contrary to other studies in which co-infections were more common (**>**60%) than single infections [14,36,32]. The reasons or the implications of this is unknown but could be due to competition for space and resources among the symbionts [38] or the tolerance of the host to harbour many bacterial communities [35]. Although this is yet to be investigated thoroughly for cassava whiteflies, but specific interactions between bacterial strains and whitefly populations was clearly evident. For example, SSA1-SG1 and SSA1-SG2 were both infected with similar strains of *Wolbachia*, which were similar to those bacteria infecting butterflies and mosquitoes, whereas SSA1-SG3 was infected with a different *Wolbachia*. Infection levels of *Rickettsia* were highest in SSA1-SG3 (54%), which was also similar to the invasive *Rickettsia* sp. nr *Bellii* strain that invaded the whitefly population MEAM1 in the USA with fitness benefits to the infected host [17]. However, infection with the same strain of *Rickettsia* in MEAM1 populations from Israel had no selective advantage to the host [39] and this further indicates specific interaction between symbiont and host genotype or the environment. When and how the *Rickettsia* invaded cassava whiteflies is unknown, but it remains to be seen if they also provide fitness benefits or not on cassava plants. Another puzzle in the jigsaw of whitefly-bacterial interactions was the detection of three different strains of *Arsenophonus* in cassava whiteflies. Strain A3 in particular was highly divergent, 7% nucleotide differences, compared to other *Arsenophonus* infecting *B. tabaci* across the world. A3 is closely related to the male killing *Arsenophonus nasoniae* [40], which again might influence the population dynamics and remains the focus of our future investigations. In summary, our findings provide insights to the diverse bacterial species infecting cassava whiteflies in African countries, and that these should be considered in future studies aiming to better understand the changing population dynamics in African cassava fields.

## Conclusions

Genetically diverse bacteria infect cassava whiteflies in Africa and their prevalence varied across the different whitefly populations and geographies. Optimising the diagnostic protocols and the characterisation of endosymbionts infecting cassava whiteflies will be highly useful for future investigations on the role of the bacteria on whitefly biology, population development and virus transmission.

## Methods

### Whitefly sampling and populations studied

Adult whiteflies collected on cassava plants in four countries; Tanzania, Uganda, Malawi and Nigeria (Table 5) and preserved in alcohol were used in diversity studies. Two laboratory populations of cassava whiteflies originally collected from Uganda and Tanzania [26] and were subsequently maintained on cassava plants in insectary conditions (27 ± 5°C, 60% relative humidity and L12:D12). These were used for detecting endosymbionts and studying their genetic diversity.

**Table 5:** **Collection sites of whitefly samples from cassava fields in Africa**

**Country**	**District (Locations)**	**Date collected**	**Number of whiteflies tested**
Tanzania	Mtwara district (Mtiniko, Namaleche)	November, 2012	10
		January, 2014	10
Tanzania	Masasi district (Napupa, Mailisita, Mnolela)	November, 2012	15
Malawi	Thyolo district (Kasonyo, Likwakwanda)	January, 2014	15
Malawi	Mulanje district (Matipwili)	January, 2014	8
Malawi	Lilongwe district (Chitedze)	January, 2014	13
Malawi	Salima district (Chitala)	November, 2013	10
Uganda	Masaka district (Masaka)	October, 2012	47
Uganda	Wakiso district (Wakiso)	October, 2012	51
Nigeria	Oyo state (Ibadan, Kajode, Ajibode, Mokola)	September, 2012	43
Nigeria	Imo state (Egbu)	October, 2012	10
Nigeria	Abia state (Umuahia)	October, 2012	15
Uganda	Namulonge (Laboratory population)	1997	----
Tanzania	Dar-es-Salaam (Laboratory population)	2010	----

### Detection and molecular characterisation of endosymbionts

Total DNA was extracted from individual adult whiteflies using the Chelex method [41] with slight modifications. Each whitefly was ground in 100 μl TE solution (10 mM Tris–HCl and 1 mM EDTA, pH 8.0) containing 20% Chelex (BIO-RAD, UK) and 300 μg Proteinase K. Samples were incubated at 60°C for 1.5 hours followed by protein denaturation at 96°C for 10 minutes. Samples were then centrifuged at 13,000 rpm and the supernatant was collected and stored at −20°C. Whitefly mtCOI genes and the endosymbiont 16S or 23S rDNA were amplified by polymerase chain reactions (PCR) using genus specific primers (see Additional file 1). New primers were designed for *Cardinium* and *Wolbachia* to increase efficiency and specificity of detection. Multilocus sequence typing (MLST) based on the diversity of five conserved housekeeping genes; *coxA*, *fbpA*, *ftsZ*, *gatB* and *hcpA* have been used as a standard tool for strain typing and evolutionary studies of *Wolbachia*. The MLST approach was used to characterize the *Wolbachia* infecting cassava whiteflies using standard primers and protocols [42]. The *Wolbachia* surface protein (*wsp*) gene was also used as an additional marker for characterisation. Amplification of these genes was carried out in 25 μl volumes using 2 μl DNA lysate as template, 0.4 μM of each primer, 0.15 mM of dNTPs, 1 × DreamTaq Green buffer and 0.5 unit DreamTaq Green DNA polymerase (Thermo Scientific Ltd., UK). Amplifications consisted of 94°C for 3 minutes followed by 38 cycles of 94°C for 30 seconds, annealing for 45 seconds (Additional file 1: Table S1), 72°C for 1.5 minutes and final extension for 7 minutes at 72°C. PCR products were visualised on 1% agarose gels containing RedSafe nucleic acid staining solution (Intron Biotechnology, Korea). PCR products were purified and submitted for Sanger sequencing (Source Bioscience, UK) in both directions per whitefly sample, and five samples were sequenced for each location. Endosymbionts were also detected and sequences from two laboratory whitefly strains (Table 5). Sequences were compared to known sequences in databases using the BLAST algorithm in NCBI.

### Developing a diagnostic tool for cassava whiteflies

The mtCOI fragments from five whitefly samples per location were sequenced, followed by phylogenetic analysis with reference sequences of haplotypes [6] for the identification of consensus haplotype groupings. The whitefly mtCOI sequences generated were analysed to identify unique restriction endonuclease sites using the software package NEBcutter (http://tools.neb.com/NEBcutter2). Three enzymes *Bgl* II (A/GATCT), *Apo* I (R/AATTY) and *Dde* I (C/TNAG) were found to produce unique patterns across SSA populations. The mtCOI fragments were re-amplified from at least 20 adults for each cassava whitefly population using 3 μl of DNA template and 1 unit of DreamTaq DNA polymerase in 30 μl volume reactions (40 cycles) for higher yields. Previously extracted DNA from four SSA2 whitefly samples were used in this assay as reference samples [26]. The RFLP was carried out in a two-step procedure. First, 15 μl of PCR products were digested with 5 units of *Bgl* II. Second, the remaining 15 μl of PCR products were digested with 5 units each of *Apo* I and *Dde* I at 37°C for 1.5 hours. Digested products were electrophoresed separately on 2% agarose gels.

### Phylogenetic and statistical analysis

The mtCOI sequences from the whitefly, the 16S or 23S rDNA sequences from the endosymbionts and the MLST sequences from *Wolbachia* were aligned separately using ClustalW of MEGA 5.2 [43]. Phylogenetic trees were constructed by the maximum-likelihood method using MEGA 5.2. Different nucleotide substitution models were used based on the lowest Bayesian information criterion scores obtained. Phylogenetic trees for mtCOI and *Wolbachia* were generated using the T93 + G + I substitution model, the HKY + G substitution model for *Arsenophonus*, the K2 + G substitution model for *Rickettsia* and the K2 substitution model for *Cardinium* [44]. The robustness of the clades was assessed by 1000 bootstrap replicates.

The probabilities of bacterial infections in cassava whitefly populations were predicted using simple binomial logistic regression. Each bacterium was used as the dependent variable and the whitefly populations as independent variables. Differences in infection patterns among groups were evaluated by Tukey’s HSD test using the glht function from multcomp package of R [45].

### Availability of supporting data

The data sets supporting the results of this article are available in the MLST and EMBL database with unique sequence and accession numbers. These are currently publicly available.

Genbank accession numbers generated in this study are as below; mtCOI sequences KM377899 to KM377952, and KM407138 to KM407141; *Wolbachia wsp* KP208705 to KP208733; *Arsenophonus* 23S rDNA KM377863 to KM377898, *Rickettsia* 16S rDNA.

KM386372 to KM38687; and *Cardinium* KM386388. The accession number for the MLST sequence types on the pubMLST database for the *Wolbachia* infecting cassava whitefly are 423–425 and 427.

## Additional file

Additional file 1: Table S1. Primer sequences and annealing temperatures used for PCR amplification. **Table S2.** Multiple comparisons of mean infection incidence of symbionts: Tukey contrasts.
