# Supplementary material for: Prevalence and genetic diversity of endosymbiotic bacteria infecting cassava whiteflies in Africa
Source: BMC Microbiol. 2015 May 2;15:93. doi: 10.1186/s12866-015-0425-5 (PMC4434523; doi:10.1186/s12866-015-0425-5)
Supplement: Additional file 1: Table S1. — Primer sequences and annealing temperatures used for PCR amplification. Table S2. Multiple comparisons of mean infection incidence of symbionts: Tukey contrasts. [file 12866_2015_425_MOESM1_ESM.pdf]

Table S1: Primer sequences and annealing temperatures used for PCR amplification

Target gene	Primer Name	Sequence (5’→3’)	Reference	Amplicon length	Annealing temperature
B. tabaci mtCOI	MTCO10<br>MTCO12	TTGATTTTTTGGTCATCCAGAAGT<br>TCCAATGCACTAATCTGCCATATTA	Frohlich *et al.*, 1999	870 bp	50℃
Portiera 16S rDNA	28F<br>1098R	TGCAAGTCGAGCGGCATCAT<br>AAAGTTCCCGCCTTATGCGT	Zchori-Fien and Brown, 2002	1050 bp	58℃
Arsenophonus 23S rDNA	Ars23S-1<br>Ars23S-2	CGTTTGATGAATTCATAGTCAAA<br>GGTCCTCCAGTTAGTGTTACCCAAC	Chiel *et al.*, 2007	750 bp	58℃
Rickettsia 16S rDNA	Rb-F<br>Rb-R	GCTCAGAACGAACGCTATC<br>GAAGGAAAGCATCTCTGC	Gottlieb *et al.*, 2006	960 bp	58℃
Wolbachia 16S rDNA	Wol16S-F<br>Wol16S-R	CGGGGGAAAAATTTATTGCT<br>CCCCATCCCTTCGAATAGGTAT	Heddi *et al.*, 1999<br>This study	730 bp	58℃
Wolbachia wsp gene	81F<br>471R	TGGTCCAATAAGTGATGAAGAAAC<br>AAAAATTAAACGCTACTCCA	Zhou *et al.*, 1998	600 bp	53℃
Cardinium 16S rDNA	Card-F<br>Card-R	TAGACACACACGAAAGTTCATGT<br>GCATGCAATCTACTTTACACTGG	This study	650 bp	57℃
Hamiltonella 16S rDNA	Hb-F<br>Hb-R	TGAGTAAAGTCTGGGAATCTGG<br>AGTTCAAGACCGCAACCTC	Gueguen *et al.*, 2010	730 bp	58℃
Fritschea 23S rDNA	Frit-F<br>Frit-R	GAGTTTGATCATGGCTCAGATTG<br>GCTCGCGTACCACTTTAAATGGCG	Gueguen *et al.*, 2010	630 bp	62℃

Table S2: Multiple comparisons of mean infection incidence of symbionts: Tukey contrasts

(p values ≤ 0.05, ≤ 0.01, ≤ 0.001 represented as ‘*’, ‘**’, ‘***’, respectively)

Arsenophonus			
Comparison		z value	Pr(>$\lvert z \rvert$)
SSA1-SG1	SSA1-SG2	-0.483	0.9885
SSA1-SG1	SSA1-SG3	5.078	<0.001***
SSA1-SG1	SSA1-SG5	2.972	0.0237*
SSA1-SG1	SSA3	1.939	0.2900
SSA1-SG2	SSA1-SG3	4.270	<0.001***
SSA1-SG2	SSA1-SG5	2.793	0.0402*
SSA1-SG2	SSA3	2.030	0.2453
SSA1-SG3	SSA1-SG5	-1.637	0.4657
SSA1-SG3	SSA3	-1.949	0.2847
SSA1-SG5	SSA3	-0.555	0.9808

Rickettsia			
Comparison		z value	Pr(>$\lvert z \rvert$)
SSA1-SG1	SSA1-SG2	2.150	0.1586
SSA1-SG1	SSA1-SG3	5.279	<0.001***
SSA1-SG1	SSA1-SG5	-0.015	1.0000
SSA1-SG1	SSA3	1.086	0.7748
SSA1-SG2	SSA1-SG3	3.210	0.0080**
SSA1-SG2	SSA1-SG5	-0.017	1.0000
SSA1-SG2	SSA3	-0.748	0.9303
SSA1-SG3	SSA1-SG5	-0.018	1.0000
SSA1-SG3	SSA3	-3.194	0.0086**
SSA1-SG5	SSA3	0.016	1.0000

Wolbachia			
Comparison		z value	Pr(>$\lvert z \rvert$)
SSA1-SG1	SSA1-SG2	3.083	0.01543*
SSA1-SG1	SSA1-SG3	-4.417	<0.001***
SSA1-SG1	SSA1-SG5	-1.163	0.75455
SSA1-SG1	SSA3	-0.659	0.96111
SSA1-SG2	SSA1-SG3	-4.605	<0.001***
SSA1-SG2	SSA1-SG5	-3.459	0.00420**
SSA1-SG2	SSA3	-3.224	0.00949**
SSA1-SG3	SSA1-SG5	2.791	0.03660*
SSA1-SG3	SSA3	2.666	0.05189
SSA1-SG5	SSA3	0.285	0.99839

Reference for supplementary material

1. Frohlich D, Torres JI, Bedford I, Markham, P, Brown J: **A phylogeographical analysis of the *Bemisia tabaci* species complex based on mitochondrial DNA markers.** *Mol Ecol* 1999, **8:**1683–91.
2. Zchori-Fein E, Brown JK: **Diversity of prokaryotes associated with *Bemisia tabaci* (Gennadius) (Hemiptera: Aleyrodidae).** *Ann Entomol Soc Am* 2002, **95:**711–718.
3. Chiel E, Gottlieb Y, Zchori-Fein E, Mozes Daube N, Katzir N, Inbar M, Ghanim M: **Biotype-dependent secondary symbiont communities in sympatric populations of *Bemisia tabaci*.** *Bull Entomol Res* 2007, **97:**407–13.
4. Gottlieb Y, Ghanim M, Chiel E, Gerling D, Portnoy V, Steinberg S, Tzuri G, Horowitz AR, Belausov E, Mozes-daube N, Kontsedalov S, Gershon M, Gal S, Katzir N, Zchori-Fien, E: **Identification and Localization of a *Rickettsia* sp . in *Bemisia tabaci* (Homoptera : Aleyrodidae).** *Appl Environ Microb* 2006, **72:**3646–52.
5. Heddi A, Grenier AM, Khatchadourian C, Charles H, Nardon P: **Four intracellular genomes direct weevil biology: nuclear, mitochondrial, principal endosymbiont, and *Wolbachia*.** *Proc Natl Acad Sci USA* 1999, **96:**6814–6819.
6. Zhou W, Neill SO: **Phylogeny and PCR-based classification of *Wolbachia* strains using wsp gene sequences.** *Proc R Soc London Ser B* 1998, **265:**509–15.
7. Gueguen G, Vavre F, Gnankine O, Peterschmitt M, Charif D, Chiel E, Gottlieb Y, Ghanim M, Zchori-Fien E, Fleury F: **Endosymbiont metacommunities, mtDNA diversity and the evolution of the *Bemisia tabaci* (Hemiptera: Aleyrodidae) species complex.** *Mol Ecol* 2010, **19:**4365–4378.
